# Endurance interval training in obese mice reduces muscle inflammation and macrophage content independently of weight loss

**DOI:** 10.14814/phy2.12012

**Published:** 2014-05-20

**Authors:** M. Constantine Samaan, Katarina Marcinko, Sarah Sikkema, Morgan D. Fullerton, Tahereh Ziafazeli, Mohammad I. Khan, Gregory R. Steinberg

**Affiliations:** 1 Division of Pediatric Endocrinology, Department of Pediatrics, Faculty of Health Sciences, McMaster University, Ontario, Canada; 2 Department of Medicine, Faculty of Health Sciences, McMaster University, Ontario, Canada

**Keywords:** Cytokines, endurance exercise, inflammation, macrophage

## Abstract

Obesity is associated with chronic low‐grade inflammation that involves infiltration of macrophages into metabolic organs such as skeletal muscle. Exercise enhances skeletal muscle insulin sensitivity independently of weight loss; but its role in regulating muscle inflammation is not fully understood. We hypothesized that exercise training would inhibit skeletal muscle inflammation and alter macrophage infiltration into muscle independently of weight loss. Wild type C57BL/6 male mice were fed a chow diet or a high‐fat diet (HFD, 45% calories fat) for 6 weeks. Then, mice maintained on the HFD either remained sedentary (HFD Sed) or exercised (HFD Ex) on a treadmill for another 6 weeks. The exercise training protocol involved conducting intervals of 2 min in duration followed by 2 min of rest for 60 min thrice weekly. Chow‐fed control mice remained sedentary for the entire 12 weeks. Muscle cytokine and macrophage gene expression analysis were conducted using qRT‐PCR, and muscle macrophage content was also measured using immunohistochemistry. Muscle cytokine protein content was quantified using a cytokine array. The HFD increased adiposity and weight gain compared to chow‐fed controls. HFD Sed and HFD Ex mice had similar body mass as well as total and visceral adiposity. However, despite similar adiposity, exercise reduced inflammation and muscle macrophage infiltration. We conclude that Endurance exercise training modulates the immune‐metabolic crosstalk in obesity independently of weight loss, and may have potential benefits in reducing obesity‐related muscle inflammation.

## Abstract (precis)

e12012

Obesity is associated with chronic low‐grade inflammation that involves infiltration of macrophages into metabolic organs such as skeletal muscle. Exercise enhances skeletal muscle insulin sensitivity independently of weight loss; but its role in regulating muscle inflammation is not fully understood. In this article, we show that endurance interval training inhibited skeletal muscle inflammation and reduced macrophage infiltration into muscle independently of weight loss in mice.

## Introduction

Consumption of high‐calorie diets and a sedentary lifestyle have resulted in a global obesity epidemic with one in six people around the world being overweight or obese, with millions of children impacted globally (Reilly 2006; de Onis et al. 2010; WHO Fact sheet). Obesity is associated with innate immune system activation and chronic low‐grade inflammation in metabolic organs (Shoelson et al. 2003). The inflammatory response is associated with enhanced lipolysis and secretion of inflammatory cytokines that originate from immune cells infiltrating the adipose tissue including monocytes. As these cells are exposed to local tissue milieu, they differentiate to inflammatory or “M1” macrophages, as opposed to anti‐inflammatory or “M2” resident tissue macrophages that are critical to maintaining tissue homeostasis. The M1 macrophages in turn secrete cytokines that propagate local tissue inflammation, promoting lipolysis and insulin resistance (Galic et al. 2010; Samaan 2011).

In addition to their role in adipose tissue, it has become evident that macrophages play an important role in inflammation of other metabolic organs including skeletal muscle (Olefsky and Glass 2010). Over the past few years, there has been increasing evidence of the presence of macrophages in skeletal muscle in obesity. In mice, the deletion of the anti‐inflammatory cytokine IL‐10 (Hong et al. 2009) or PPAR*γ* (Hevener et al. 2007), the latter being a master regulator of adipogenesis and muscle metabolism, results in enhanced macrophage infiltration into muscle. In obese humans, macrophages have been detected in skeletal muscle and macrophage content correlates negatively with insulin sensitivity (Varma et al. 2009; Fink et al. 2013). Macrophages in muscle mainly exist in the intermyocellular fat depot and rarely between the myofibrils (Weisberg et al. 2003; Hong et al. 2009). Another intriguing location that they inhabit is the adipose tissue at the muscle‐fat junction, which may suggest a bidirectional migration between the two tissues and not though the vasculature (Di Gregorio et al. 2005; Patsouris et al. 2009; Varma et al. 2009). In contrast to the above studies, several reports documented the absence of macrophages from muscle, or the presence of macrophages at very low levels (Xu et al. 2003; Bruun et al. 2006; Tam et al. 2012). As muscle takes up the majority of meal‐related carbohydrate intake, muscle insulin resistance is a critical determinant of glycemia and contributes to the development of type 2 diabetes once insulin demand exceeds pancreatic supply (Dandona et al. 2004). Therefore, it is important to understand the role of macrophage‐muscle crosstalk as this may pave the way to define interventions that help combat muscle inflammation.

One of the main prescribed interventions in obesity management is exercise, which can improve insulin sensitivity in the absence of weight loss in both mice and humans (Bruce et al. 2006; Ringseis et al. 2011). In terms of its effects on the immune system, exercise reduces the number of circulating inflammatory monocytes (Timmerman et al. 2008) and monocyte infiltration into adipose tissue, as well as diminishing the phenotypic switch of macrophages to a more inflammatory phenotype (Kawanishi et al. 2010). Exercise also increases regulatory T‐lymphocytes (Wang et al. 2012), and these responses by both innate and adaptive immune system arms ultimately lead to suppression of inflammation. It has been shown that endurance exercise reduces TNF*α* and IL‐6 gene expression in muscle in frail elderly participants, which may be mediated via reduced expression of toll‐like receptor 4 (Lambert et al. 2008). However, whether chronic exercise alters skeletal muscle macrophage content or inflammatory status is not currently known. Therefore, we tested the hypothesis that chronic exercise training reduces skeletal muscle inflammation in obese mice by reducing macrophage infiltration or by altering macrophage phenotype.

## Methods

### Animal experiments

Wild type male C57BL/6 mice were housed in ventilated cages and maintained on a 12‐h light/dark cycle (*n *=**5–8 per condition). At 6 weeks of age, mice were maintained on control chow diet (17% kcal from fat; Diet 8640, Harlan Teklad, Madison, WI) or switched to a high‐fat diet (45% calories fat, Research Diets, New Brunswick, NJ) for 12 weeks as previously described (Fullerton et al. 2013b). Body weights were monitored weekly.

#### Exercise training protocol

For the first 6 weeks of the HFD, mice remained sedentary in their cages. Mice were then randomly divided into two groups; one that remained sedentary (HFD Sed) while the other group underwent exercise training (HFD Ex) for the last 6 weeks of the 12‐week HFD intervention. The chow‐fed mice remained sedentary over the entire 12 weeks. The HFD Ex mice were familiarized with the treadmill the week before starting the exercise protocol, running at 10–15 m/min for 15 min for 3 days. Each exercise training session involved treadmill running for 2 min followed by 2‐min of active rest for 60 min thrice weekly. Running speed started at 16 m/min (~80% of maximal exercise capacity) and was increased by 1 m/min per week.

This exercise training protocol is similar to a recent study (Hoydal et al. 2007) that found that aerobic interval training in mice maximizes training adaptations while minimizing potential injuries. Importantly, this exercise protocol was also designed to maximize training adaptations while avoiding potential weight loss, which can result from long duration, low‐intensity treadmill (Marques et al. 2010) or wheel running (Zhou et al. 2005). This latter point was essential when designing the exercise protocol, as adiposity is the most important predictor of macrophage inflammatory status (Vieira et al. 2009).

#### Assessment of body composition

At 11 weeks, computed tomography (CT) scans were conducted to assess total adiposity of lean and obese mice, as recently described (Galic et al. 2011) with minor modification. Briefly, mice were anesthetized and images were acquired on an X‐SPECT (Gamma Medica, Northridge, CA). Calibration in Hounsfield units (HU) was achieved by including a water‐filled tube in each scan. Amira software (version 5.2.2; Visage Imaging, Berlin, Germany) was used to analyze CT images. Whole body adipose tissue content was measured using a computer‐assisted application selecting a range of volume from −450 to −125 HU. To determine the visceral adiposity, the mouse midsection region was isolated by clipping that scan at the bottom of the thoracic cage and at the top of the pelvis. The adipose tissue was isolated from the abdominal tissue, and subcutaneous adipose tissue was manually separated from visceral adipose tissue.

#### Measurement of glucose, insulin, and HOMA‐IR

For blood glucose and serum insulin, mice were anesthetized using isofluorane (Pharmaceutical Partners of Canada Inc.; Richmond Hill, Ontario, Canada), and blood samples were collected from the facial vein 24 h post exercise in the fed and fasting states (12 h overnight fast). Blood glucose was checked using the Accu‐Check Aviva blood glucose meter (Roche Diagnostics; Mannheim, Germany). For serum isolation, whole blood was allowed to clot for 30 min and the sample was spun for 7 min at 3800*g* and serum was stored at −80°C until further use. The Rat/Mouse Insulin ELISA kit from Millipore (St. Charles, MO) was used to measure serum insulin according to manufacturer's instructions. HOMA‐IR was calculated using the following equation: fasting glucose (mmol/L) × fasting insulin (*μ*U/L)/22.5 (Andrikopoulos et al. 2008).

#### Glucose tolerance tests

Mice were fasted for 6 h and then underwent glucose tolerance testing (intraperitoneal injection of 1 g/kg glucose) 24 h after their training bout (Beck Jorgensen et al. 2009). Blood was collected from tail vein at baseline, 20, 40, 60, 90, and 120 min following glucose administration. Blood glucose was checked using the Accu‐Check Aviva blood glucose meter.

#### Tissue collection

Mice were sacrificed 72 h after the last bout of exercise and gastrocnemius muscle was snap frozen in liquid nitrogen and stored at −80°C until further use. Gastrocnemius muscle (10 mg) was powdered in liquid nitrogen, and this muscle was used for all analyses unless otherwise indicated.

### Analytical methods

#### RNA isolation and gene expression analysis

Powdered muscle was added to 1 mL of Trizol. The sample was then homogenized in Trizol and RNA isolation was completed using Qiagen RNAeasy mini kits (Qiagen, Valencia, CA) following manufacturer's instructions. cDNA generation was performed using 1 *μ*g of RNA with SuperScript^®^ III reverse transcriptase kit (Invitrogen, Carlsbad, CA). Quantitative real‐time PCR was conducted using TaqMan^®^ Gene Expression Assays (Applied Biosystems; Foster City, CA) to measure the expression of F4/80, CD11c, CD206, chemokine C‐C ligand 2 (CCL2), Interleukin 1β (IL‐1β), interleukin 6 (IL‐6), tumor necrosis factor *α* (TNF*α*), and interleukin 10 (IL‐10). TATA box binding protein (TBP) was used as a reference gene when measuring cytokine gene expression in muscle, and 36B4 marker was used as a reference gene for macrophages. All samples were analyzed using the Rotor‐Gene 6000 (Corbett Research; Mortlake, Australia) and genes expressed relative to reference genes using the ΔΔCt method (Livak and Schmittgen 2001). Muscle triglycerides were measured as previously described (Fullerton et al. 2013b).

#### Immunohistochemistry

To assess muscle macrophage content further, paraffin‐embedded muscle was sectioned and after dewaxing with xylene, rehydration in ethanol gradient was performed. This was followed by antigen retrieval with 10 mmol/L sodium citrate (pH 6.5) for 15 min, and 1% fetal calf serum and 3% hydrogen peroxide in phosphate buffered saline were used to quench endogenous peroxidase. Tissue slides were then blocked with 5% normal rabbit serum (40 min), Avidin D (Biotin/Avidin Blocking Kit, Vector Laboratories; Burlinghame, CA) (15 min), and Biotin (Biotin/Avidin Blocking Kit, Vector Laboratories) (15 min).

For antigen detection, a 2‐h incubation with rat anti‐mouse F4/80 (1:100) (AbD Serotec, Oxford, UK) was followed by a 1‐h incubation in biotin‐conjugated secondary antibody (1:50) (Vector Laboratories), and then completed with a 30‐min incubation in VECTASTAIN ABC solution system (Vector Laboratories). To develop the slides, DAB Substrate Kit (Vector Laboratories) was used with hematoxylin counterstaining. Sections were imaged using Nikon Eclipse 90i microscope and images analyzed to determine macrophage content using NIS Element 64 bit 3.22.11 Software (Nikon Inc., Melville, NY). The area for each section (*n *=**4 per condition) was measured when each section was scanned, by assigning four side limitation and pictures were taken. The final number of images per section ranged from 250 to 400. Macrophages were counted manually at 400× magnification, and the number of macrophages was normalized to the section area. The researcher counting the cells was blinded to the experimental group allocations.

#### Muscle Bio‐Plex assay

Bio‐Plex Pro 23‐Plex mouse cytokine kit (Bio‐Rad) was used to measure muscle cytokine levels in powdered gastrocnemius muscle as per manufacturer's instructions with minor modifications (*n *=**4 per condition). Briefly, muscle tissue was washed three times in wash buffer, and put in ice‐cold Bio‐Plex cell lysis buffer containing factors 1 and 2 from the tissue lysis kit, PMSF, and one complete mini protease inhibitor cocktail tablets (Roche Applied Science) per 50 mL of buffer. The tissue preparation was transferred to an ice‐cold tissue homogenizer and dounced 25 times on ice. The sample was transferred to a new tube and placed at −80°C for 1 h. This was followed by sonication (Tekmar Sonic Disrupter, Cincinnati, OH) of the tissue on ice for 20 sec for five times at 50% power. The extracts were collected by centrifugation at 3400*g* at 4°C for 30 min, and the samples were transferred to a new tube and aliquoted at −80°C until further processing. The samples were thawed only once at the time of processing. Standard curves were generated and cytokine measurements were normalized to total protein content of the sample and expressed as pg *μ*L^−1^ *μ*g^−1^ protein.

#### Statistical analysis

Data were analyzed with Excel and GraphPad Prism 6.0 for Windows (GraphPad Software, San Diego, CA). Analysis of variance (ANOVA) was used to perform analyses on the data from chow, high‐fat fed sedentary, and exercise groups and Tukey post hoc test was done to determine the differences between groups. *T*‐test was used to compare dietary intake data and exercise capacity. Kruskal–Wallis test was used for area under the curve calculations. A *P*‐value of 0.05 and below is considered statistically significant. Data are presented as mean ± SEM.

## Results

**Figure 1.**
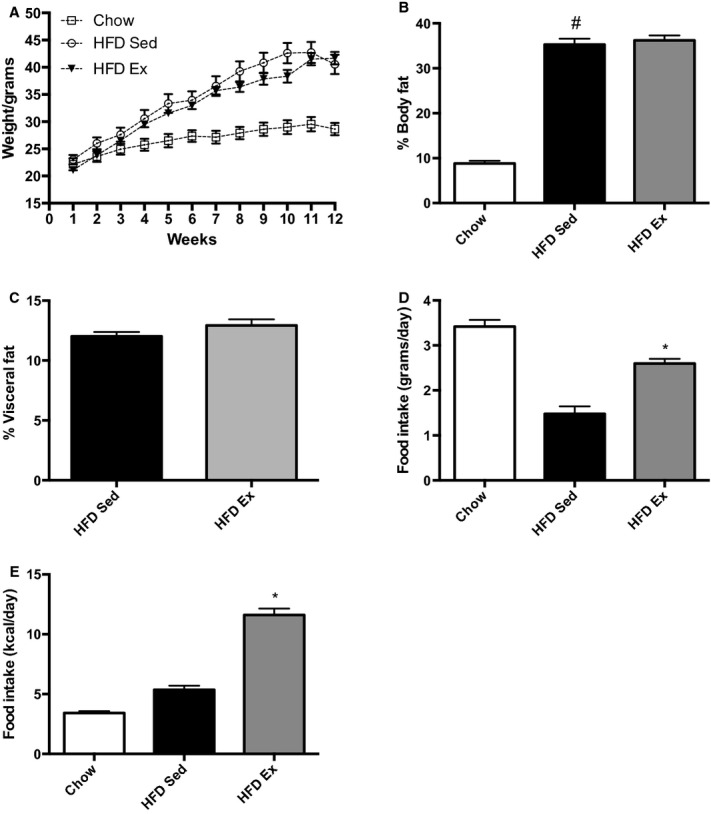
Effect of chronic endurance interval training on (A) body weight, (B) total adiposity, (C) visceral adiposity, (D) amount of food intake (g/day), and (E) caloric intake in mice. Weight was measured weekly and % adiposity was measured on CT scans. *P *<**0.01 for comparison between chow and high‐fat fed sedentary mice. ^#^Statistical significance for comparisons between chow and HFD Sed groups.

Mice fed the HFD gained significantly more weight compared to chow‐fed controls and had significant increases in adiposity (*P* < 0.001) (Fig. 1A). Importantly, both HFD Sed and HFD Ex mice gained similar weight and demonstrated similar total and visceral adiposity, allowing us to compare the effect of exercise independently of differences in body mass or adiposity (Fig. 1A–C). The weight gain and adiposity in HFD fed mice was comparable to recent studies from our laboratory using the same dietary conditions (Fullerton et al. 2013a; Jorgensen et al. 2013). HFD Ex mice consumed more food and had higher caloric intake than sedentary mice (*P *<**0.0001) (Fig. 1D and E), which has been noted previously in studies using C57BL/6 mice in exercise studies (Brownlow et al. 1996). As anticipated, the exercise training also elicited an increase in maximal exercise capacity with improved maximal time to exhaustion (16.8 ± 2.6 vs. 29.1 ± 4.6 min; *t*‐test, *P* =0.003) and maximal speed at exhaustion (16.0 ± 1.4 vs. 22.2 ± 2.2 m/min; *t*‐test, *P* = 0.003) in the HFD Ex group pre‐ and post exercise training, respectively.

**Figure 2.**
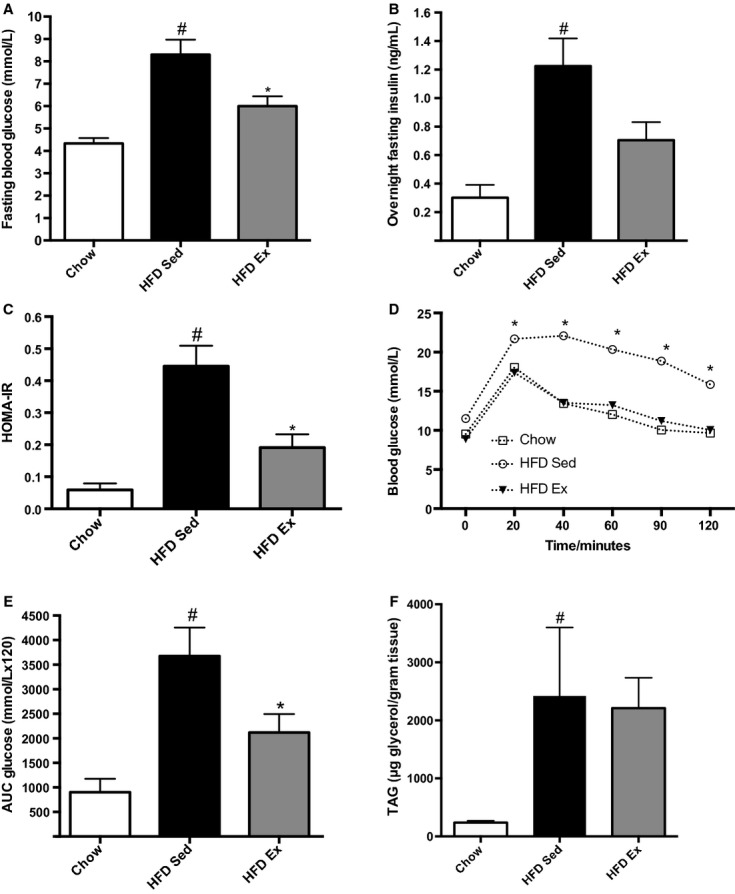
Exercise training improves whole body glucose tolerance and insulin sensitivity. (A) Fasting blood glucose and (B) serum insulin. (C) HOMA‐IR calculated from the fasting blood glucose and serum insulin. (D) Glucose tolerance and (E) area under the curve. ^#^Statistical significance for comparisons between chow and HFD Sed groups. *Statistical significance for differences between HFD Sed and HFD Ex groups.

To determine the effect of exercise training on glucose homeostasis, we measured fasting glucose and insulin levels and performed glucose tolerance tests. Fasting glucose (*P *=**0.0001), fasting insulin (*P *=**0.001), and HOMA‐IR (*P *≤**0.0001) were significantly elevated in HFD Sed mice compared to chow controls (Fig. 2A–C). Importantly compared to HFD Sed mice, exercise training lowered fasting glucose (*P *=**0.012) and HOMA‐IR (*P *=**0.004), and improved glucose tolerance (Fig. 2D and E).

A HFD has been shown to increase muscle triglycerides (Kraegen et al. 1991), and exercise training in chow‐fed mice has also been shown to increase this parameter, a condition known as the athletes’ paradox (Goodpaster et al. 2001). We found that muscle triglyceride level was dramatically elevated by the HFD but exercise did not lead to further increases (Fig. 2F). These data suggest that changes in muscle inflammation are not related to alterations in muscle triglyceride levels.

These data established that our exercise protocol elicited expected changes in glucose tolerance and insulin sensitivity.

The primary aim of our study was to assess the chronic effects of exercise training on muscle inflammation; therefore, we assessed markers of muscle inflammation primarily regulated by macrophages 48 h after the last exercise bout. This time point is important as it alleviates concerns that observed changes are directly related to the acute effects of exercise.

**Figure 3.**
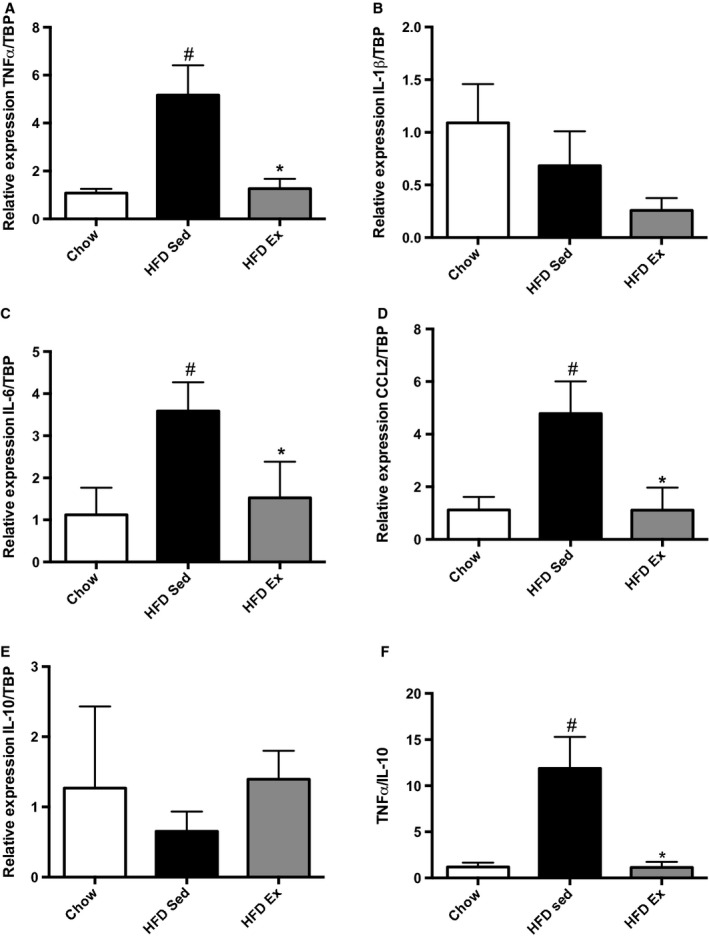
Exercise training effects on skeletal muscle cytokine mRNA expression. (A–E) Gene expression was normalized to TBP; (F) TNF*α*/IL‐10 ratio in chow, HFD Sed, and HFD Ex. ^#^Statistical significance for comparisons between chow and HFD Sed groups. *Statistical significance for differences between HFD Sed and HFD Ex groups.

We found that TNF*α*, IL‐6, and CCL2 (also known as monocyte chemotactic protein‐1 [MCP‐1]) expression were increased in HFD Sed mice compared to chow‐fed controls (Fig. 3). There were no differences in IL‐1*β* or IL‐10 expression (Fig. 3). Importantly, HFD Ex mice demonstrated significant downregulation of inflammatory cytokine gene expression when compared with HFD Sed mice, characterized by lower TNF*α* (*P *=**0.007), IL‐6 (*P *=**0.03), and CCL2 (*P *=**0.004), while IL‐1*β* expression was not different (*P *=**0.3) (Fig. 3). In addition, the gene expression of IL‐10, a known anti‐inflammatory cytokine tended to be higher in HFD Ex mice compared to sedentary controls (*P *=**0.09) (Fig. 3F). The ratio of TNF*α*/IL‐10 gene expression, which may be considered a measure of the pro‐ to anti‐inflammatory phenotype of the muscle, was increased in HFD Sed mice compared to chow controls and restored in muscle of HFD Ex mice (Fig. 3F, *P *=**0.01).

**Figure 4.**
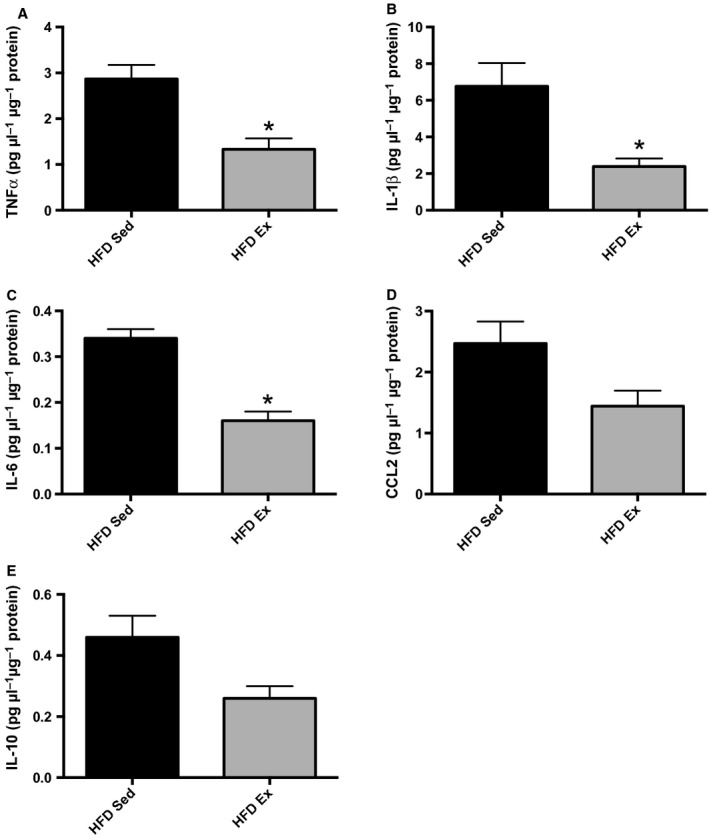
(A–E) Exercise training effects on skeletal muscle cytokine protein levels. Protein concentrations were normalized to sample protein content. *Statistical significance for differences between HFD Sed and HFD Ex groups.

To examine whether exercise training‐induced changes in mRNA translated to changes in protein expression, we conducted a bead‐based cytokine array in muscles from HFD Sed and HFD Ex trained mice (Fig. 4; Table 1). HFD Ex mice had significantly reduced levels of TNF*α* (Fig. 4A, *P *=**0.03), a trend in reduction in IL‐1*β* (Fig. 4B, *P *=**0.08) and reduced IL‐6 (Fig. 4C, *P *=0.002), while the chemokine CCL2 trended lower (Fig. 4D, *P *=**0.06). Surprisingly, the expression of the anti‐inflammatory cytokine IL‐10 tended to be reduced in HFD Ex mice compared to HFD Sed controls (Fig. 4E, *P *=**0.13). Many other inflammatory cytokines and chemokines with less defined functions in obesity were also reduced in muscle of HFD Ex mice as detailed in Table 1.

**Table 1.** Modulation of muscle cytokine levels by exercise.

Cytokine	HFD Sed	SEM	HFD Ex	SEM	*P*‐value
IL‐1*α*	0.571	0.199	0.223	0.037	0.41
IL‐2	0.621	0.155	0.271	0.036	0.27
IL‐3	0.072	0.013	0.033	0.009	0.09
IL‐4	0.039	0.006	0.02	0.004	0.09
IL‐5	0.153	0.016	0.078	0.012	0.03
IL‐9	3.557	0.799	1.663	0.553	0.18
IL‐10	0.46	0.065	0.26	0.039	0.13
IL‐12(p40)	0.406	0.037	0.238	0.016	0.04
IL‐12(p70)	2.2	0.389	0.717	0.116	0.05
IL‐13	4.979	0.514	2.653	0.407	0.11
IL‐17	0.149	0.018	0.086	0.014	0.09
Eotaxin	50.667	7.468	13.627	3.588	0.03
G‐CSF	7.759	2.974	2.876	1.228	0.28
GM‐CSF	5.933	1.01	2.615	0.439	0.1
IFN*γ*	0.389	0.046	0.171	0.018	0.03
CXCL1	0.722	0.135	0.361	0.063	0.14
CCL3	1.1	0.128	0.477	0.06	0.03
CCL4	0.509	0.117	0.589	0.162	0.9
CCL5	0.152	0.013	0.086	0.022	0.2

The *P*‐values reported are for HFD Sed versus HFD Ex mice. Statistical significance is set at ≤0.05.

**Figure 5.**
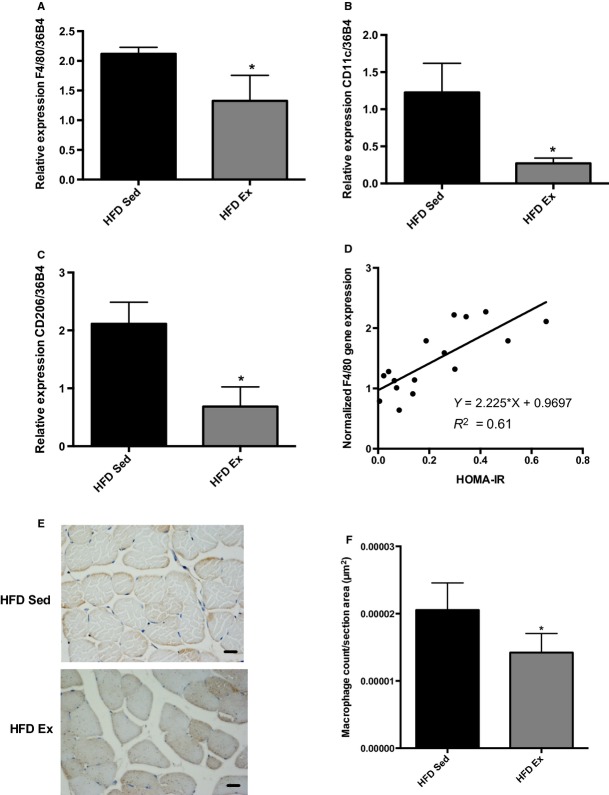
Changes in muscle macrophage content and phenotype by exercise. (A–C) Gene expression was normalized to 36B4: F4/80, *P* = 0.009; CD11c, *P* = 0.02; CD206, *P* = 0.01. (D) Correlation between HOMA‐IR and macrophage content. (E) Immunohistochemistry images using F4/80 antibody to detect macrophages in muscle of HFD Sed and HFD Ex mice. (F) Quantification of immunohistochemistry images for macrophage content of HFD Sed and HFD Ex mice (*P* = 0.04). *Statistical significance for differences between HFD Sed and HFD Ex groups.

On the basis of the evidence that macrophages infiltrate skeletal muscle in obesity and its content correlate with insulin resistance (Varma et al. 2009; Fink et al. 2013), we investigated whether the reduced inflammation noted in the HFD Ex group was correlated with altered muscle macrophage content. In HFD Ex mice, the total muscle macrophage content declined as noted by gene expression analysis of F4/80 (*P *=**0.009, Fig. 5A). This reduction in muscle macrophage infiltration was due to reduced muscle content of both inflammatory (CD11c, *P *=**0.025, Fig. 5B) and anti‐inflammatory (CD206, *P *=**0.015, Fig. 5C) macrophages; importantly, muscle macrophage content correlated positively with HOMA‐IR (Fig. 5D). Immunohistochemical analysis confirmed this result, and revealed that macrophages were present between muscle fibers, intermyocellular fat and around blood vessels, and were reduced proportionately from these regions with exercise (*P *=**0.04, Fig. 5E and F).

## Discussion

In this study, chronic endurance interval training of obese mice resulted in a substantial improvement in muscle inflammation independent of weight loss or reduction in adiposity. Reductions in muscle inflammation involved reductions in muscle macrophage content. The reduction in muscle macrophages correlated strongly with improvements in HOMA‐IR. This is concordant with studies in humans showing muscle macrophage content to be positively correlated with body mass index and insulin resistance (Varma et al. 2009).

Previous studies have shown that combined aerobic and resistance exercise training and not low‐calorie diet alone in elderly subjects reduces muscle IL‐6 and TNF*α* gene expression (Lambert et al. 2008). Consistent with these results we found lower levels of TNF*α* mRNA and protein in HFD exercise trained mice. TNF*α* has been shown to disrupt muscle insulin sensitivity by activating serine threonine stress kinases (De Alvaro et al. 2004; Plomgaard et al. 2005), inducing the suppressor of cytokine signaling 3 (SOCS3) (Jorgensen et al. 2013) and by inhibiting fatty acid metabolism (Steinberg et al. 2006). TNF*α* is primarily derived from macrophages (Weisberg et al. 2003; Fain 2006) suggesting that the large reductions in TNF*α* following exercise training is likely mediated through either reduced muscle macrophage infiltration or reduced macrophage inflammation.

IL‐6 expression was reduced in muscle by exercise training. Under resting conditions, such as those studied in the current set of experiments which were conducted 48 h after the last exercise bout, the amount of IL‐6 released from muscle is very low and its primary source of production is thought to be macrophages (Pedersen and Febbraio 2012). In contrast, during acute exercise IL‐6 expression is transiently increased and released from skeletal muscle, an effect that is dependent on activation of c‐Jun terminal kinase and activation of the transcription factor AP‐1 (Whitham et al. 2012). Importantly, the production of muscle‐derived IL‐6 quickly subsides following exercise (Whitham et al. 2012). Therefore, our observation of reduced IL‐6 expression with exercise training is consistent with the reduction in other macrophage‐derived cytokines.

Another important cytokine implicated in the development of insulin resistance is IL‐1*β* (Masters et al. 2010). We found that exercise training tended to reduce IL‐1*β* mRNA expression, but the effects on protein expression were much more dramatic, with IL‐1*β* expression being reduced by approximately 70% compared to HFD Sed controls. IL‐1*β* mRNA expression is under the control of the transcription factors NF‐*κ*B, PU.1, and HIF1*α*, while protein expression and cleavage is dependent on activation of the NLRP3 inflammasome (Masters et al. 2010). Future studies examining how exercise training might reduce muscle NLRP3 inflammasome activation are warranted.

We found that exercise training reduced the expression of CCL2, an important factor controlling migration of monocytes to muscle (Sartipy and Loskutoff 2003; Kamei et al. 2006). In addition to acting as a chemokine, CCL2 has also been shown to directly cause insulin resistance (Sartipy and Loskutoff 2003; Kamei et al. 2006). Several other molecules that are less well studied in muscle metabolism and exercise also emerged on our cytokine assays. For example, IL‐12 is produced by monocytes and dendritic cells and stimulate the differentiation of naïve T‐lymphocytes to Th1 cells, and stimulates TNF*α* and IL‐8 production by neutrophils (Al‐Mohanna et al. 2002). In addition, IL‐17 acts as a chemokine recruiting monocytes and neutrophils to inflamed tissues, and reduces IL‐12 mediated IFN*γ* production in mononuclear cells by downregulation of IL‐12R*β*2 subtype (Miossec et al. 2009; Toh et al. 2009).

An important question which our study has attempted to address was whether the reduction in muscle inflammatory cytokines was due to direct manipulation of muscle macrophage inflammation or was indirectly related to reduced muscle macrophage content. Our data show that this chronic exercise regimen reduces total muscle macrophage content (F4/80 marker), and this is the result of reduced M1 (CD11c marker) and M2 (CD206 marker) macrophage complement. This is an intriguing result and is in contrast to the acute effects of exercise, which show an increase in M2 load in muscle (Ikeda et al. 2013), and argues for reduced macrophage content and inflammatory cytokines as the main mechanism through which this exercise regimen mediate its effects.

Chronic exercise is known to reduce the number of circulating inflammatory monocytes (Timmerman et al. 2008), which will provide a smaller pool of cells that are available to migrate to tissues; this is coupled with reduced production of several known monocyte chemoattractants by muscle as shown in our study, with lower CCL2, CCL3, and IL‐17. In addition, this exercise regimen may reduce the differentiation of monocytes to M1 phenotype as it suppresses IFN*γ*, a known factor that polarizes monocytes to M1 phenotype, and downregulate M2 markers like CD206 that we measured (Gordon and Martinez 2010).

In our study, IFN*γ* is reduced with exercise in muscle (Table 1). Despite the suppression of IFN*γ*, which we predicted will increase M2 polarization this was not the case. The differentiation of macrophage to the M2 phenotype requires a cytokine mix that is dominated by IL‐4 (Stein et al. 1992) and IL‐13 (Doyle et al. 1994). The reduced M2 cell number in muscle with this exercise regimen is coupled with a trend to lower levels of IL‐4, while IL‐13 levels did not differ significantly between groups. While we did not measure circulating monocyte numbers, reduced muscle chemoattractants and lower production of critical cytokines driving M1 macrophage differentiation (IFN*γ*) and the lack of a rise in cytokines that drive M2 phenotype (IL‐4, IL‐13) all explain the macrophage centric phenotype noted in this study and the reduced inflammatory responses noted. Our results argue for a more profound effect of this chronic exercise routine on muscle‐macrophage crosstalk that has not been previously appreciated, as the majority of studies have evaluated acute exercise regimens on macrophages in muscle (Ikeda et al. 2013).

In summary, we demonstrate that chronic endurance interval training downregulate muscle inflammation and macrophage content independently of changes in adiposity. This reduction in muscle macrophage content appears to be a potentially a key contributing factor in reducing muscle inflammation following chronic exercise training. Thus, this exercise training regimen represents a potential method by which obesity‐related muscle inflammation can be mitigated.

## Acknowledgments

We would like to acknowledge the help of Ms. Lesley Wiltshire with performance of Bio‐Plex assay.

## Conflict of Interest

None declared.
